# Modeling the Binding of Anticancer Peptides and Mcl-1

**DOI:** 10.3390/ijms25126529

**Published:** 2024-06-13

**Authors:** Shamsa Husain Ahmed Alhammadi, Bincy Baby, Priya Antony, Amie Jobe, Raghad Salman Mohammed Humaid, Fatema Jumaa Ahmed Alhammadi, Ranjit Vijayan

**Affiliations:** 1 Department of Biology, College of Science, United Arab Emirates University, Al Ain P.O. Box 15551, United Arab Emirates; 2 The Big Data Analytics Center, United Arab Emirates University, Al Ain P.O. Box 15551, United Arab Emirates; 3 Zayed Center for Health Sciences, United Arab Emirates University, Al Ain P.O. Box 15551, United Arab Emirates

**Keywords:** Mcl-1, BH3 domain, anti-cancer peptides, BH3 mimetics, protein–protein interactions, molecular dynamics

## Abstract

Mcl-1 (myeloid cell leukemia 1), a member of the Bcl-2 family, is upregulated in various types of cancer. Peptides representing the BH3 (Bcl-2 homology 3) region of pro-apoptotic proteins have been demonstrated to bind the hydrophobic groove of anti-apoptotic Mcl-1, and this interaction is responsible for regulating apoptosis. Structural studies have shown that, while there is high overall structural conservation among the anti-apoptotic Bcl-2 (B-cell lymphoma 2) proteins, differences in the surface groove of these proteins facilitates binding specificity. This binding specificity is crucial for the mechanism of action of the Bcl-2 family in regulating apoptosis. Bim-based peptides bind specifically to the hydrophobic groove of Mcl-1, emphasizing the importance of these interactions in the regulation of cell death. Molecular docking was performed with BH3-like peptides derived from Bim to identify high affinity peptides that bind to Mcl-1 and to understand the molecular mechanism of their interactions. The interactions of three identified peptides, E2gY, E2gI, and XXA1_F3dI, were further evaluated using 250 ns molecular dynamics simulations. Conserved hydrophobic residues of the peptides play an important role in their binding and the structural stability of the complexes. Understanding the molecular basis of interaction of these peptides will assist in the development of more effective Mcl-1 specific inhibitors.

## 1. Introduction

The pro-survival protein myeloid cell leukaemia-1 (Mcl-1), a member of the Bcl-2 family, is among the most frequently upregulated genes in cancer [1]. Analysis of genomic data from The Cancer Genome Atlas (TCGA) indicated high Mcl-1 protein expression in several cancer types [2], including lung [2,3], breast [4,5], colon [6,7], ovarian carcinomas [8], gastric [9,10], multiple myeloma [11], non-small-cell lung cancer [12,13], and malignant melanoma [14]. 

Bcl-2 proteins modulate apoptosis, a mechanism of programmed cell death that regulates homeostasis. These proteins feature a shared Bcl-2 homology (BH) domain and a carboxy-terminal transmembrane domain [15]. The transmembrane domain is key for mitochondrial localization [16], while the BH domains (BH1–BH4) [17] regulate protein–protein interactions [18]. Bcl-2 proteins are categorized into antiapoptotic proteins (Mcl-1, Bcl-2, Bcl-extra-large (Bcl-XL), BFL-1/Bcl-2-related protein A1 (Bcl-2A1), Bcl-B, and Bcl-W), multidomain pro-apoptotic executioner proteins (BAX, BAK, and BOK), and BH3-only pro-apoptotic proteins (BIM, BAD, Noxa, PUMA, HRK, and BMF) [19]. 

Mcl-1 inhibits apoptosis by heterodimerizing with pro-apoptotic Bcl-2 members via its BH3 domain and is upregulated in several cancers, including lung [2,3], breast [4,5], colon [6,7], ovarian carcinoma [8], and gastric [9,10] cancer, mediating resistance to apoptosis induced by conventional chemotherapy and targeted therapy [3,20]. Undesirable side effects, drug resistance, and limited selectivity of traditional anti-cancer approaches prompt the exploration of non-conventional cancer therapeutics, namely, peptide-based therapeutics. Such an approach exploits the activity of anti-cancer peptides (ACPs), which have been reported to outperform established therapies in terms of specificity, sensitivity, and lower toxicity in therapeutic applications [21,22]. ACPs are a class of peptides typically spanning 10–60 amino acids with anti-tumor activity and minimal susceptibility to drug resistance [22]. These peptides have been exploited in combination therapy to enhance tumor sensitivity to chemotherapy [15]. The low production cost, target specificity, ease of synthesis and modification, low toxicity, and high tissue penetration make ACPs promising candidates for anti-cancer treatment [23,24].

The BH3-binding groove of Mcl-1 carries four pockets (P1–P4) that interact with hydrophobic side chains (H1–H4) of pro-apoptotic proteins [25,26]. These pockets and the Arg263 residue within the Mcl-1 BH3 groove represent hotspots crucial for peptide binding [27]. Recently, Wang et al. (2021) reported the Mcl-1 hotspot residues within the P1–P4 pockets based on the Mcl-1/BIM complex (PDB: 2NL9)—P1: Leu235, Leu246, and Val249; P2: Met231, Met250, Val253, Phe254, Leu267, Phe270, Gly271, Val274, Leu290, and Ile294; P3: His224, Ala227, Phe228, and Thr266; and P4: Val216, Val220, and Val265 [28]. 

Through library screening of stabilized α-helices of Bcl-2 domains, Stewart et al. (2010) reported the Mcl-1 BH3 helix as a unique inhibitor of the Mcl-1 inhibitor and a sensitizer for apoptosis [29]. Additionally, a conserved salt bridge was observed between Arg263 of Mcl-1 and an Asp residue of BH3-only proteins [29,30]. Selective Mcl-1 inhibitors, some of which are under clinical trial, were shown to occupy the P1–P4 pockets and interact with Arg263 of Mcl-1 [1,28]. For instance, S63845 [25] and AZD599 [31] exhibit strong salt bridge interactions with Arg263, while AMG-176 [32,33] and A-1210477 engage in hydrogen bonding with Arg263 [34]. 

The development of specific inhibitors for anti-apoptotic proteins, particularly through the use of BH3 α-helical peptides that mimic interactions with hydrophobic binding pockets of anti-apoptotic proteins, has been a significant area of research. Stewart et al. demonstrated the potent and selective binding of Mcl-1 BH3 α-helix peptides to Mcl-1, confirming the specificity of these interactions [29]. Rezaei et al. further showed that BH3 peptides derived from Bim specifically bind to the binding groove of Mcl-1 [35]. The study aimed to elucidate the interaction of different Bim peptides with Mcl-1 at the molecular level by compiling a set of Bim-based peptides from the literature and screening them against Mcl-1. Protein–protein docking was conducted to find optimal docking conformations based on cluster size, pose energy, and interactions. A set of Bim-based peptides (Supplementary Table S1), identified from the literature, were screened against Mcl-1 [36,37,38,39,40,41,42]. The length of these peptides varied from 18 to 26 amino acids. This study highlights the binding dynamics and affinity of peptides to Mcl-1, especially in the context of developing novel therapeutic strategies for cancer treatment.

## 2. Results

A set of BH3 peptides derived from Bim was compiled from the literature and screened against Mcl-1 using protein–protein docking. The binding affinity of Bim-based anticancer peptides to the Mcl-1 anti-apoptotic protein was assessed using PIPER [43]. These resulting structures were then grouped into clusters and ranked according to cluster size, and the largest clusters were prioritized. The pose with the best fit was selected for each protein–peptide complex based on cluster size. The most populated clusters likely represent the most biologically relevant conformations of the peptide–protein complexes. 

### 2.1. Binding of E2gI, E2gY, and XXA1 F3dI with Mcl-1

**Figure 1:**
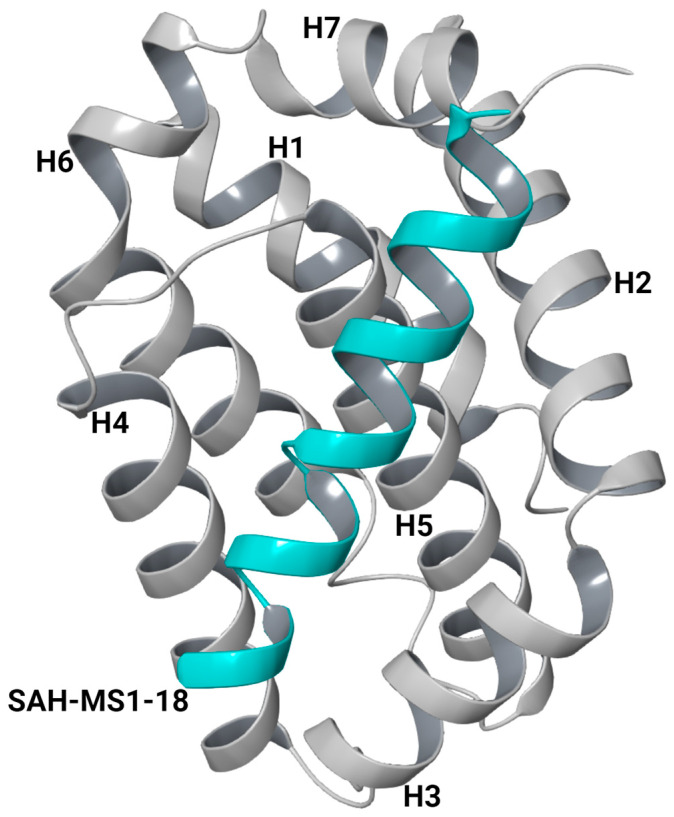
Structure of Mcl-1 (grey) with a modified Bim BH3 peptide, SAH-MS1-18 (cyan) (PDB: 5W89) [35].

**Figure 2:**
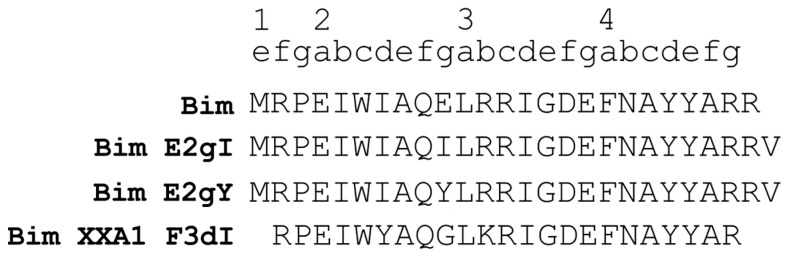
Sequence of the top scoring Bim-based peptides. The heptad convention used to refer to positions in the BH3 peptide is shown. Numbering uses the convention (abcdefg)n. Complete heptad repeats 2 to 4 are indicated above the sequences.

**Figure 3:**
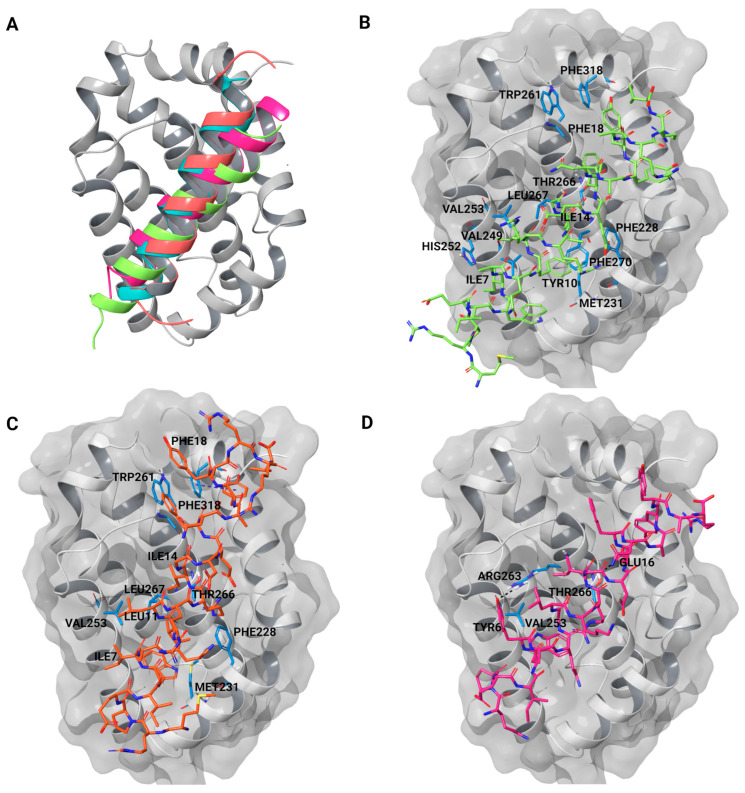
(**A**) The crystal structure of Mcl-1 (grey), complexed with a modified Bim BH3 peptide SAH-MS1-18 (cyan) (PDB ID:5W89) [35] as well as docked E2gI (orange), E2gY (green), and XXA1 F3dI (pink). (**B**) Docked pose of E2gY in Mcl-1. (**C**) Docked pose of E2gI in Mcl-1. (**D**) Docked pose of XXA1 F3dI in Mcl-1. Mcl-1 residues are colored in blue.

Three Bim-based peptides, E2gI, E2gY, and XXA1 F3dI, were shortlisted from the protein–protein docking analysis. These peptides were derived from the BH3 motif of pro-apoptotic Bim and demonstrated strong binding to Mcl-1, particularly within the hydrophobic groove. In contrast, their interactions with Bcl-XL, another anti-apoptotic protein, showed a lower binding score and weaker binding in the analysis (Supplementary Table S2). This differential binding affinity highlights the specificity of these peptides for Mcl-1 over Bcl-XL. The binding mode of the peptides was measured based on the number of clusters that formed in a protein–peptide docking using PIPER. E2gY, E2gI, and XXA1 F3dI were observed to bind in the hydrophobic BH3-binding groove with better binding scores (Table 1 and Supplementary Table S3). The large cluster size observed for the binding of E2gY, E2gI, and XXA1 F3dI peptides to Mcl-1 indicates a potentially favorable and stable interaction within the hydrophobic groove. Mcl-1 has a helical core structure composed of eight α-helices (α1–α8). The amphipathic α5 is surrounded by the other helices, creating a hydrophobic groove where the BH3 peptide binds. Helices including α2, α3, and α4 contribute to the formation of the BH3-binding groove, while α5 and α8 form the base of the groove (Figure 1). The BH3-domain pro-apoptotic proteins contain four highly conserved hydrophobic residues at positions 2d, 3a, 3d, and 4a as per the heptad notation (Figure 2). The docked poses were aligned to resemble the binding pose of the peptide inhibitor, SAH-MS1-18, observed in the crystallographic structure of Mcl-1 (PDB ID: 5W89) (Figure 3A). This alignment serves to confirm the binding mode of the peptides, indicating that they likely interact with the target molecule in a similar manner as the known inhibitor. The cluster size and pose energy for these interactions are detailed in Table 1.

The binding of E2gI and E2gY exhibited similarities, with a notable difference at position 3g; E2gI had Ile10, which was substituted by a tyrosine (Tyr10) in E2gY. Both peptides interacted with the canonical BH3-binding groove, engaging helices α3, α4, α5 (BH1), α8 (BH2), and α2 (BH3), as expected. Conserved hydrophobic residues at positions 2d, 3a, 3d, and 4a interacted with Mcl-1 in a manner similar to the corresponding residues of the Bim peptide inhibitor in the crystallographic structure (Figure 3). The conserved residue Ile7 at position 2d interacted with Val249 and His252 in the E2gY/Mcl-1 complex. This interaction is significant because it contributed to the stabilization of the peptide within the hydrophobic binding groove of Mcl-1. Similar to the interaction involving Leu210, the interactions of Ile7 with Val249 and His252 are crucial for potently inhibiting the anti-apoptotic function of Mcl-1 [44]. The residue Leu11 at position 3a in E2gY and E2gI was the most significant residue for the complex’s stability. It interacted with Val249, His252, Val253, and Leu267 in the E2gY/Mcl-1 complex, while in the case of E2gI, it interacted with Val253. Another conserved hydrophobic residue at position 3d (Ile14) of each Bim peptide has been indicated to be important for stabilizing the complex [44]. Ile14 of E2gY formed strong interactions with the surrounding residues Thr266, Leu267, Phe228, Met231, and Phe270 of Mcl-1. Ile14 of E2gI showed hydrophobic interactions with Thr266 and Leu267. The conserved Phe18 at 4a showed hydrophobic interactions with Trp261 and Phe318 in E2gI, and in E2gY, Phe18 interacted with Val220, Val216, Val265, Thr266, and Phe319. Specifically, Ile10 in E2gI and Tyr10 in E2gY were involved in interactions with the hydrophobic groove of Mcl-1. Ile10 interacted with Phe228, Met231, Thr266, and Leu267, whereas Tyr10 in E2gY interacted with Met231, Val249, and Phe270. In comparison with the other two peptides, XXA1 F3dI showed a smaller cluster size and less pose energy. The conserved residue at 2d (Tyr6) formed a hydrogen bond with Arg263 and hydrophobic interactions with Val253. Phe17 at position 4a of XXA1 F3dI formed an interaction with Val253. Another conserved residue, Glu16 at the 4f position, formed hydrogen bond interactions with Thr266 (Table 1).

### 2.2. Molecular Dynamics Simulations of the Docked Poses

The top three Bim-based peptide complexes, determined by the best cluster size, were subjected to molecular dynamics (MD) simulations to study the structural dynamics of the complexes. MD simulations were performed for 250 ns to evaluate the stability of the complex and the bound peptide. Analysis of the simulation trajectories showed that the simulations were able to effectively refine the peptide-binding pose. This refinement indicates that the dynamic behavior of the system during the simulation allowed for a more accurate representation of the interaction of the peptides with the target molecule. 

**Figure 4:**
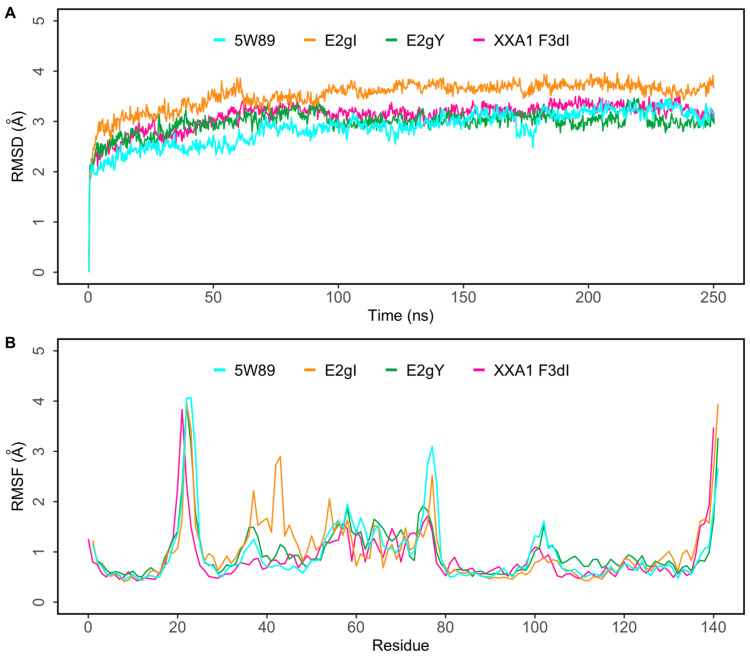
(**A**) Root mean square deviation (RMSD) of protein Cα atoms obtained from 250 ns simulations of the crystal structure (PDB ID: 5W89) with bound SAH-MS1-18 (cyan) and docked E2gI (orange), E2gY (green), and XXA1 F3dI (pink). (**B**) Root mean square fluctuation (RMSF) of protein residues obtained from 250 ns simulations. Crystal structure (PDB ID: 5W89) with bound SAH-MS1-18 (cyan) and docked E2gI (orange), E2gY(green), and XXA1 F3dI (pink).

The root mean square deviation (RMSD) of a protein in a simulation provides insight into the overall deviation from the initial structure. Figure 4A shows the progression of protein RMSD in the presence of peptides in 250 ns MD simulations. In all peptide complexes, Mcl-1/E2gI, Mcl-1/E2gY, and Mcl-1/XXA1 F3dI, the system stabilized under 4 Å after a few nanoseconds (Figure 4A). After an initial period of adjustment, the peptides reached a relatively stable conformation during the simulation. To investigate the residue-level protein flexibility for each system, the root mean square fluctuation (RMSF) values of backbone atoms were evaluated. Figure 4B illustrates the fluctuations of each residue in the protein structure. Based on the calculated RMSF values, as depicted in Figure 4B, it was noted that the loop region spanning from Ala190 to Thr205, which connected the α1 and α2 helices, exhibited the largest fluctuations. Furthermore, the flexibilities of the loop regions (Asp236 to Asp242) in all the complexes may be directly related to the structural adjustment of Mcl-1 helix H4 with the three peptides (Figure 4B). The radius of gyration (Rg) shows the structural compactness and stability of the molecules [45]. The Rg values of E2gI/Mcl-1, E2gY/Mcl-1, and XXA1 F3dI/Mcl-1, and the crystallographic structure with the peptide inhibitor (PDB ID: 5W89), were calculated from the generated MD trajectories of 250 ns (Supplementary Figure S1).

**Figure 5:**
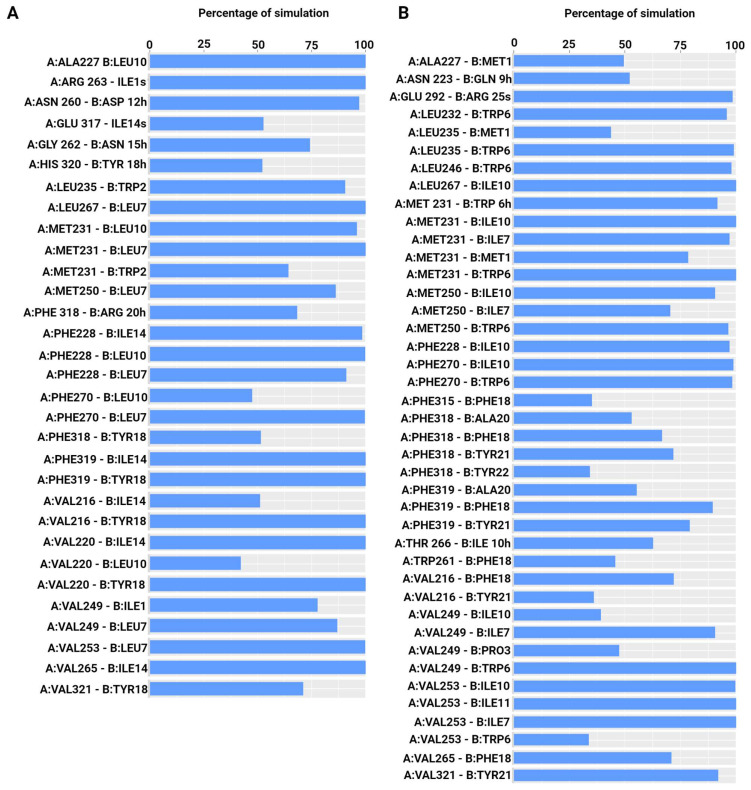
Percentage of simulation time during which intermolecular polar and hydrophobic contacts were retained between Mcl-1 and peptides in the 250 ns systems. (**A**) Mcl-1/SAH-MS1-18 inhibitor, and (**B**) Mcl-1/E2gI.

**Figure 6:**
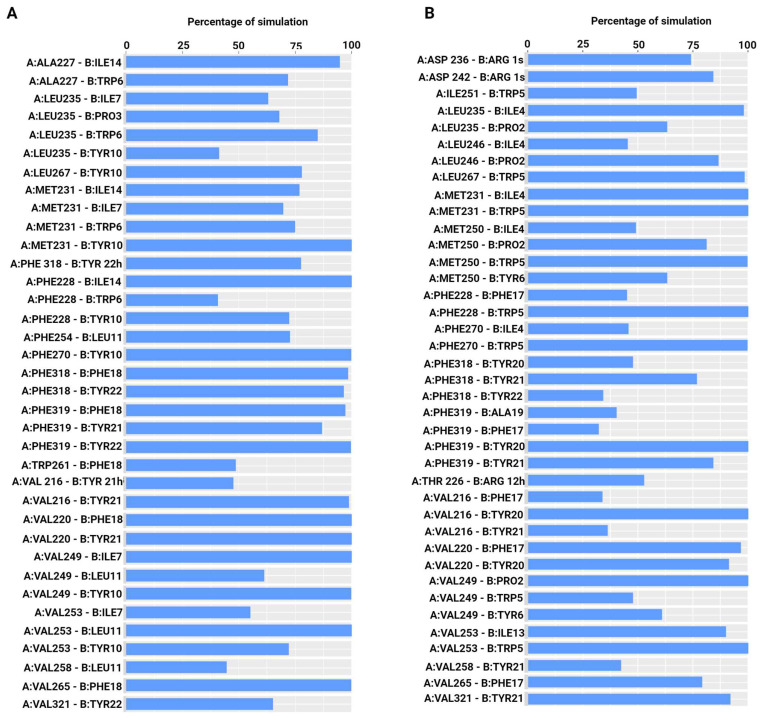
Percentage of simulation time during which intermolecular polar and hydrophobic contacts were retained between Mcl-1 and peptides in the 250 ns systems. (**A**) Mcl-1/E2gY, and (**B**) Mcl-1/XXA1 F3dI.

Peptides bound strongly to the binding site throughout the simulations. The RMSF of the peptides was also analyzed. A low level of fluctuation was observed for all the peptides, indicating stable binding (Supplementary Figure S2). MD simulation trajectories were analyzed to find conserved hydrophobic and hydrophilic interactions between the peptides and Mcl-1 (Figure 5 and Figure 6). Comparative analysis of the interactions of these peptides with Mcl-1 and the crystallographic protein was performed to identify the role of specific residues that might govern the binding affinity of these peptides towards Mcl-1. In MD simulations, the four conserved hydrophobic residues at positions 2d (Ile7), 3a (Leu11), 3d (Ile14), and 4a (Phe18) of E2gI and E2gY formed stable interactions with the hydrophobic residues of the binding groove formed by the BH3 regions of Mcl-1 (Figure 5B and Figure 6A). In the MD simulations, the four conserved hydrophobic residues at positions 2d (Ile7), 3a (Leu11), 3d (Ile14), and 4a (Phe18) of E2gI and E2gY had stable interactions with the hydrophobic residues of the binding groove formed by the BH3 regions of Mcl-1.

Interactions that persisted between the peptides and Mcl-1 for at least 50% of the simulation time were examined. The conserved residue at position 2d (Ile7) interacted with Met231, Met250, Val249, and Val253 in the E2gI/Mcl-1 complex, while in the case of E2gY, Ile7 interacted with Met231, Leu235, Val249, and Val253 throughout the simulation. Position 3a of the peptides (Leu11) was crucial for complex stability. It primarily interacted with Val253 in the E2gI/Mcl-1 complex, and in the E2gY/Mcl-1 complex, Leu11 interacted with Val249, Val253, Val258, and Phe254. Another conserved hydrophobic residue at position 3d (Ile14) of each Bim peptide played an important role in stabilizing the complex. IIle14 of E2gY formed strong interactions with the surrounding residues Ala227, Phe228, and Met231 of Mcl-1. The conserved Phe18 at 4a showed hydrophobic interactions with Phe315, Phe318, Phe319, Val216, and Val265 in E2gI, and in E2gY, Phe18 interacted with Phe318, Phe319, Val220, and Val265 (Figure 5B and Figure 6A). The three conserved hydrophobic residues (2d, 3a, and 3d) contributed to the structural stability of the complex; they were necessary for the high binding affinities to Mcl-1. The residue at position 3g (Ile10 in E2gI and Tyr10 in E2gY) was also involved in important interactions. Ile10 interacted with Phe228, Met231, Met250, Val253, Leu267, and Phe270, whereas Tyr10 interacted with Phe228, Met231, Leu235, Val249, Val253, Leu267, and Phe270. The conserved residue at 2d (Tyr6) interacted with Val249 and Met250. Another conserved hydrophobic residue at position 3d (Ile13) of XXA1 F3dI interacted with Val253, and the conserved Phe17 at 4a interacted with Val220, Val265, and Phe228 of Mcl-1 (Figure 6B).

To gain deeper insights into the concerted motions of the docked complexes observed in the MD simulations, principal component analysis (PCA) was performed on the peptide-bound simulation trajectories. The PCA results revealed that the first two principal components (PC1 and PC2) described the majority of the significant concerted motions observed in the simulations [46]. It highlights the critical conformational states and transitions that underpin the successful binding of the peptide to the protein. In PC1, significant changes were observed in the Mcl-1 binding interface, predominantly involving helices α3 and α4, and leading to a transition towards a more open conformation, which enhanced peptide binding. The peptides appeared to slide and adjust within the binding site in PC1. In PC2, a translational motion was exhibited in α1. Helix α1 was not close to the binding region and was not involved in binding. PC1 and PC2 of all the peptide-bound trajectories, including the co-crystallized peptide and the docked peptides, exhibited the same concerted motions described above, indicating that the Bim-based peptides bound and interacted in a manner similar to the co-crystallized inhibitor.

## 3. Discussion

Myeloid cell leukemia-1 (Mcl-1) is an anti-apoptotic protein that engages in heterodimerization with the proapoptotic Bcl-2 members to inhibit apoptotic cell death [47]. Structural studies have shown that, while there is high overall structural conservation among the anti-apoptotic Bcl-2 proteins, differences in the surface groove of these proteins facilitate binding specificity. This binding specificity is crucial for the mechanism of action of the Bcl-2 family in regulating apoptosis. The surface groove in Mcl-1 and other anti-apoptotic Bcl-2 proteins serves as a binding site for the BH3 domain of proapoptotic proteins, thereby preventing their pro-death functions and promoting cell survival [28,48]. 

Development of specific inhibitors for anti-apoptotic proteins, particularly through the use of BH3 α-helical peptides that mimic interactions with hydrophobic binding pockets of anti-apoptotic proteins, has been a significant area of research. The BH3 domain is a conserved region found in both pro- and anti-apoptotic members of the Bcl-2 family. BH3-only proteins promote apoptosis by interacting with anti-apoptotic Bcl-2 family members, releasing pro-apoptotic proteins and initiating the apoptotic cascade. Mimicking these interactions with synthetic peptides offers a promising strategy for disrupting the function of anti-apoptotic proteins [49,50]. Bim, a pro-apoptotic protein, plays a crucial role in regulating cell death pathways by interacting with anti-apoptotic proteins like Mcl-1. Studies have shown that peptides derived from Bim can disrupt the Mcl-1/Bim complex, leading to apoptosis [51,52]. The binding affinity between Bim peptides and Mcl-1 has been investigated, with results indicating that Bim BH3 peptides exhibit strong interactions with Mcl-1. 

Based on the data obtained in this study, three Bim-based peptides, E2gI, E2gY, and XXA1_F3dI, were identified to have high affinity for Mcl-1 through protein–protein docking analysis. The peptides bound in the hydrophobic BH3-binding groove with large cluster size. The BH3 domain pro-apoptotic proteins contained four highly conserved hydrophobic residues at positions 2d, 3a, 3d, and 4a, as per the heptad notation (Figure 2). These residues play a crucial role in mediating interactions with the BH3-binding groove of anti-apoptotic proteins like Mcl-1 [29,40,53,54,55,56]. The conserved residue Ile7 at position 2d interacted with Val249 and His252 in the E2gY/Mcl-1 complex. This interaction is significant because it contributed to the stabilization of the peptide within the hydrophobic binding groove of Mcl-1. Several studies have highlighted the significance of hydrophobic residues within the binding pocket of Mcl-1. Beekman and Howell (2015) reported on the significance of targeting specific residues such as Leu210, Val249, and His252 to disrupt the anti-apoptotic function of Mcl-1. Inhibitors that interact with these key residues could disrupt the function of Mcl-1, which is crucial for promoting cell survival [57]. Joseph et al. optimized stapled BH3 peptides as potent Mcl-1 inhibitors. They demonstrated that effective binding of stapled peptides was achieved through interactions between the hydrophobic staple and the hydrophobic patches on the surface of Mcl-1. This interaction relies on the complementary nature of hydrophobic residues, creating a stable interface between the peptide and Mcl-1, and enhancing the overall affinity of the complex [58]. The interaction with conserved residues at 2d (Ile7), 3a (Leu11), and 3d (Ile14) of Bim peptides has been shown to be important for stabilizing the complex [44]. Previous studies by Stewart et al. and Parikh et al. have highlighted the significance of conserved hydrophobic residues in Mcl-1 BH3 peptides [29,56]. Substitution of these residues by alanine resulted in a loss of affinity of the Mcl-1 BH3 α-helix for Mcl-1, underscoring their importance in peptide binding and anti-apoptotic function. Foight et al. engineered three peptides, MS1, MS2, and MS3, derived from the BH3 domain of the pro-apoptotic protein Bim. These peptides demonstrated notable specificity and affinity for the hydrophobic groove of Mcl-1, as evidenced in BH3 profiling assays [38]. Specifically, Ile10 in E2gI and Tyr10 in E2gY were involved in interactions with the hydrophobic groove of Mcl-1. 

MD simulations were performed for 250 ns to evaluate the stability of the complex. In MD simulations, the four conserved hydrophobic residues at positions 2d (Ile7), 3a (Leu11), 3d (Ile14), and 4a (Phe18) of the E2gI and E2gY were also found to be stable and consistent during the simulations, indicating that they contributed strongly to the binding of the peptide in the hydrophobic groove. The study highlights the binding dynamics and affinity of peptides to Mcl-1, especially in the context of developing novel therapeutic strategies for cancer treatment.

## 4. Materials and Methods

### 4.1. Preparation of the Protein

The three-dimensional structure of Mcl-1 (PDB ID: 5W89) complexed with the modified Bim BH3 peptide SAH-MS1-18 was downloaded from the Protein Data Bank (PDB). The bound peptide structure was used as a control in this study. The protein structure was pre-processed using the Protein Preparation Wizard tool within the Schrödinger Suite 2022-4 [59]. During the pre-processing step, the protein structure was optimized and refined to correct any structural irregularities, such as missing atoms, incorrect bond angles, or steric clashes. Following the pre-processing step, the protein structure was described using the OPLS 2005 force field. The optimized protein structure was subjected to energy minimization to further refine the geometry and ensure structural stability [60].

### 4.2. Preparation of Peptides

A set of BH3-like peptides derived from Bim peptides was compiled from the literature and screened to isolate high-affinity Bim-based peptides to Mcl-1. The tertiary structure of the peptides was downloaded from the Apoptosis-Inducing Anticancer Peptides Database (ApInAPDB) (Supplementary Table S2) [61].

### 4.3. Protein–Protein Docking

The prepared structure of Mcl-1 and the peptides were docked using protein–protein docking in the BioLuminate module of the Schrödinger software suite [62]. One structure is treated as the receptor and the other as the ligand. Protein–protein docking was performed using the PIPER (Protein–Protein Interaction Property Similarity) program, which utilizes the Fast Fourier Transform (FFT) correlation approach to return near-native conformations of the docked poses. By employing various scoring functions and optimization techniques, PIPER efficiently explores the conformational space of protein complexes to predict their most stable configurations. During the docking process, PIPER employs a grid-based approach to locate the best poses of the two protein structures with a maximum resolution in the poses of about 5°. The docking is performed as a rigid-body optimization, meaning that there is no subsequent minimization of the interfacial region [43]. The docked poses were ranked based on PIPER cluster size.

### 4.4. Molecular Dynamics Simulation

The function of most proteins depends on their dynamics. Therefore, it is necessary to explore the dynamics of the docked complexes to assess the intermolecular contacts and the stability of the Mcl-1 protein and the Bim peptide complexes. Hence, MD simulations of the docked complexes were carried out for 250 ns to assess the dynamics and stability of the best binding conformation of the three peptides shortlisted based on the cluster size [63]. The MD simulations were run using Desmond with the OPLS-AA 2005 force field. The complexes were placed in an orthorhombic box of size 64 Å × 64 Å × 64 Å and solvated with single-point-charge water molecules using the Desmond System Builder [64]. The simulation system was neutralized with the required number of counterions, and the salt concentration was set at 0.15 M NaCl. Prior to running MD simulations, all systems were subjected to the steepest descent minimization and Desmond’s default eight-stage relaxation protocol. The electrostatic interactions were calculated using the Particle Mesh Ewald (PME) method with 1.0 nm short-range electrostatic and van der Waals cutoffs [65]. An NPT ensemble with the temperature at 300 K and the pressure at 1 atm was applied. The Nose–Hoover thermostat and the isotropic Martyna–Tobias–Klein barostat were used to maintain a temperature of 300 K and a pressure of 1 atm, respectively [66,67]. A time-reversible reference system propagator algorithm (RESPA) integrator was used with an inner time step of 2.0 fs and an outer time step of 6.0 fs [68]. Following the simulations, the root mean square deviation (RMSD) of the protein, the root mean square fluctuation (RMSF) of both the protein and the peptide, and the intermolecular interactions were computed. All bonds involving hydrogen atoms were constrained using the M-SHAKE algorithm implemented in Desmond [69]. Principal component analysis (PCA) was performed on the MD trajectories to reduce the dimensionality of the simulation data and to identify the predominant modes of motion within the system [70]. Packaged and custom scripts were used to analyze the simulation data. 

## 5. Conclusions

Mcl-1 is a promising therapeutic target in the treatment of cancer. Peptides derived from the BH3 region of pro-apoptotic proteins have demonstrated the ability to bind to the hydrophobic groove of anti-apoptotic Mcl-1, thereby modulating apoptotic pathways in living cells. Understanding the molecular interactions underlying protein–peptide interactions is crucial for the development of potent and specific inhibitors targeting Mcl-1. Protein–protein docking analysis of Bim-based peptides was carried out to identify high-affinity peptides that bind to Mcl-1. The results indicate that the interactions of E2gY, E2gI, and XXA1 F3dI peptides with Mcl-1 were stronger compared to other peptides studied here. Molecular dynamics simulations of Mcl-1 in complex with the three Bim-based peptides were also performed. These simulations provided insights into the structural dynamics and stability of the complexes formed between Mcl-1 and the peptides. Importantly, interactions involving conserved hydrophobic residues of the peptides were identified to play pivotal roles in maintaining the structural stability of these complexes. Understanding the structural basis of the interactions between these Bim-based peptides and Mcl-1 provides valuable insights for the development of novel therapeutic strategies for targeting the Bcl-2 family of proteins in cancer treatment.

## Figures and Tables

**Table 1:** Top-ranking peptides identified from PIPER protein–protein docking.

Peptide	PIPE Cluster Size	PIPER Pose Energy (kcal/mol)	Hydrogen Bonds ^1^	Hydrophobic Interaction ^1^	π-π ^1^	π-Cation ^1^
E2gY	455	−978.97		A:LYS234–B:TRP6 A:LEU235–B:TRP6 A:VAL249–B:TRP6 A:VAL249–B:ILE7 A:HIS252–B:ILE7 A:MET231–B:TYR10 A:PHE270–B:TYR10 A:VAL249–B:TYR10 A:VAL253–B:LEU11 A:VAL249–B:LEU11 A:HIS252–B:LEU11 A:LEU267–B:LEU11 A:THR266–B:ILE14 A:LEU267–B:ILE14 A:PHE228–B:ILE14 A:MET231–B:ILE14 A:PHE270–B:ILE14 A:THR266–B:PHE18 A:VAL265–B:PHE18 A:VAL220–B:PHE18 A:VAL216–B:PHE18 A:PHE319–B:PHE18 A:PHE319–B:TYR22 A:PHE318–B:TYR22		
E2gI	306	−886.21		A:MET231–B:MET1 A:ALA227–B:MET1 A:MET231–B:TRP6 A:VAL249–B:TRP6 A:MET231–B:MET1 A:ALA227–B:MET1 A:MET231–B:ILE10 A:THR266–B:ILE10 A:LEU267–B:ILE10 A:PHE228–B:ILE10 A:MET231–B:ILE10 A:Val253–B:LEU11 A:THR266–B:ILE14 A:THR266–B:ILE14 A:LEU267–B:ILE14 A:PHE318–B:PHE18 A:TRP261–B:PHE18 A:PHE318–B:TYR21 A:PHE319–B:TYR21 A:PHE318–B:TYR22 A:MET231–B:TRP6 A:VAL249–B:TRP6 A:PHE270–B:TRP6	A:PHE318–B:TYR21	A:HIS224–B:ARG13
XXA F3dI	304	−840.14	A:ARG263–B:TYR6 A:THR266–B:GLU16	A:PHE318–B:PHE17 A:HIS252–B:PRO2 A:PHE319–B:TYR20 A:VAL216–B:TYR20 A:LYS234–B:ILE4 A:VAL249–B:TRP5 A:VAL253–B:TRP5 A:MET250–B:TRP5 A:LEU267–B:TRP5 A:PHE270–B:TRP5 A:VAL253–B:TYR6		

^1^ Chain A represents Mcl-1, and chain B represents the bound peptide.

## Data Availability

Data sharing is not applicable to this article.

## Supplementary Materials

The supporting information can be downloaded at: https://www.mdpi.com/article/10.3390/ijms25126529/s1. References [36,37,38,39,40,41,42] are cited in the Supplementary Materials.
