# Supplementary material for: Modeling the Binding of Anticancer Peptides and Mcl-1
Source: Int J Mol Sci. 2024 Jun 13;25(12):6529. doi: 10.3390/ijms25126529 (PMC11203456; doi:10.3390/ijms25126529)
Supplement: Supplementary file 1 [file ijms-25-06529-s001.zip › ijms-2964652-supplementary.pdf]

Modeling the binding of anticancer peptides and Mcl-1

Shamsa Husain Ahmed Alhammadi[1, †], Bincy Baby[1, †], Priya Antony[1], Amie Jobe[1], Raghad Salman Mohammed Humaid[1], Fatema Jumaa Ahmed Alhammadi[1] and Ranjit Vijayan[1,2,3*]

[1] Department of Biology, College of Science, United Arab Emirates University, PO Box 15551, Al Ain, United Arab Emirates
[2] The Big Data Analytics Center, United Arab Emirates University, PO Box 15551, Al Ain, United Arab Emirates.
[3] Zayed Center for Health Sciences, United Arab Emirates University, PO Box 15551, Al Ain, United Arab Emirates.
† These authors contributed equally to this work
* Corresponding authors: ranjit.v@uaeu.ac.ae (R.V.)

Supplementary Materials

Table S1. List of peptides used in this study

Peptide	Sequence	Reference
Bim	RPEIWIAQELRRIGDEFNAYYAR	[36]
Bim A2eT-E2gG	RPEIWITQGLRRIGDEFNAYYAR	[37]
Bim A2eT-F4aI	RPEIWITQELRRIGDEINAYYAR	[38]
Bim A2eT-I2dM	RPEIWMTQELRRIGDEFNAYYAR	[38]
Bim A2eT-I3dL	RPEIWITQELRRLGDEFNAYYAR	[38]
Bim E3gK	MRPEIWIAQELRRIGDKFNAYYAR	[39]
Bim F4aE	MRPEIWIAQELRRIGDEENAYYAR	[39]
Bim F99E	IWIAQELRRIGDEENAYY	[40]
Bim F99Q	IWIAQELRRIGDEQNAYY	[40]
Bim FA1	RPEIWIAQELRRAGDVLNAYYAR	[36]
Bim FD1	RPEIWLAQYLRRLGDQINAYYAR	[36]
Bim FD2	RPEIWMAQVLRRFGDLLNAYYAR	[36]
Bim FW1	RPEIWIAQGLRRIGDTWNAYYAR	[36]
Bim I2dA	MRPEIWAAQELRRIGDEFNAYYAR	[39]
Bim I2dY	MRPEIWYAQELRRIGDEFNAYYAR	[39]
Bim I3dF	MRPEIWIAQELRRFGDEFNAYYAR	[39]
Bim I86E	EWIAQELRRIGDEFNAYY	[40]
Bim I95A	IWIAQELRRAGDEFNAYY	[40]
Bim Q90E	IWIAEELRRIGDEFNAYY	[40]
Bim XXA1	RPEIWYAQGLKRFGDEFNAYYAR	[41]
Bim XXA1 F3dI	RPEIWYAQGLKRIGDEFNAYYAR	[41]
Bim XXA1 G2gE	RPEIWYAQELKRFGDEFNAYYAR	[41]
Bim XXA1 K3bR	RPEIWYAQGLRRFGDEFNAYYAR	[41]
Bim XXA1 Y2dI	RPEIWIAQGLKRFGDEFNAYYAR	[41]
Bim XXA1 Y4eK	RPEIWYAQGLKRFGDEFNAYKAR	[41]
Bim XXA4	RPEIWYAQWLKRFGDQFNAYYAR	[41]
Bim Y103K	IWIAQELRRIGDEFNAYK	[40]
Bim Y4eK- 18	IWYAQGLKRFGDEFNAYK	[41]
Bim Y4eK- 21	RPEIWYAQGLKRFGDEFNAYK	[41]
Bim 2A	DMRPEIWIAQEARRIGDEANAYYARR	[42]
Bim A2eE	MRPEIWIEQELRRIGDEFNAYYARRV	[37]
Bim A2eF	MRPEIWIFQELRRIGDEFNAYYARRV	[37]
Bim A2eG	MRPEIWIGQELRRIGDEFNAYYARRV	[37]
Bim A2eH	MRPEIWIHQELRRIGDEFNAYYARRV	[37]
Bim A2eI	MRPEIWIIQELRRIGDEFNAYYARRV	[37]
Bim A2eK	MRPEIWIKQELRRIGDEFNAYYARRV	[37]

Bim A2eL	MRPEIWILQELRRIGDEFNAYYARRV	[37]
Bim A2eN	MRPEIWINQELRRIGDEFNAYYARRV	[37]
Bim A2eP	MRPEIWIPQELRRIGDEFNAYYARRV	[37]
Bim A2eQ	MRPEIWIQQELRRIGDEFNAYYARRV	[37]
Bim A2eR	MRPEIWIRQELRRIGDEFNAYYARRV	[37]
Bim A2eS	MRPEIWISQELRRIGDEFNAYYARRV	[37]
Bim A2eT	MRPEIWITQELRRIGDEFNAYYARRV	[37]
Bim A2eV	MRPEIWIVQELRRIGDEFNAYYARRV	[37]
Bim A2eW	MRPEIWIWQELRRIGDEFNAYYARRV	[37]
Bim A2eY	MRPEIWIYQELRRIGDEFNAYYARRV	[37]
Bim E2gA	MRPEIWIAQALRRIGDEFNAYYARRV	[37]
Bim E2gD	MRPEIWIAQDLRRIGDEFNAYYARRV	[37]
Bim E2gF	MRPEIWIAQFLRRIGDEFNAYYARRV	[37]
Bim E2gG	MRPEIWIAQGLRRIGDEFNAYYARRV	[37]
Bim E2gH	MRPEIWIAQHLRRIGDEFNAYYARRV	[37]
Bim E2gI	MRPEIWIAQILRRIGDEFNAYYARRV	[37]
Bim E2gK	MRPEIWIAQKLRRIGDEFNAYYARRV	[37]
Bim E2gN	MRPEIWIAQNLRRIGDEFNAYYARRV	[37]
Bim E2gP	MRPEIWIAQPLRRIGDEFNAYYARRV	[37]
Bim E2gQ	MRPEIWIAQQLRRIGDEFNAYYARRV	[37]
Bim E2gR	MRPEIWIAQRLRRIGDEFNAYYARRV	[37]
Bim E2gS	MRPEIWIAQSLRRIGDEFNAYYARRV	[37]
Bim E2gT	MRPEIWIAQTLRRIGDEFNAYYARRV	[37]
Bim E2gW	MRPEIWIAQWLRRIGDEFNAYYARRV	[37]
Bim E2gY	MRPEIWIAQYLRRIGDEFNAYYARRV	[37]
Bim EgV	MRPEIWIAQVLRRIGDEFNAYYARRV	[37]
Bim L3aD	MRPEIWIAQEDRRIGDEFNAYYARRV	[37]
Bim L3aE	MRPEIWIAQEERRIGDEFNAYYARRV	[37]
Bim L3aH	MRPEIWIAQEHRRIGDEFNAYYARRV	[37]
Bim L3aK	MRPEIWIAQEKRRIGDEFNAYYARRV	[37]
Bim L3aN	MRPEIWIAQENRRIGDEFNAYYARRV	[37]
Bim L3aQ	MRPEIWIAQEQRRIGDEFNAYYARRV	[37]
Bim L3aR	MRPEIWIAQERRRIGDEFNAYYARRV	[37]
Bim L3aS	MRPEIWIAQESRRIGDEFNAYYARRV	[37]

Table S2. Docking results of E2gI, E2gY, and XXA1 F3dI with Bcl-XL

Peptide	Cluster size	Pose energy (kcal/mol)
E2gI	295	-112.96
E2gY	210	-143.71
XAA1 F3dI	587	-155.49

Table S3: Docking results of other peptides with Mcl-1

Peptide	Cluster size	Pose energy (kcal/mol)
Bim A2eT-E2gG	32	-660.156
Bim A2eT-F4aI	300	-1004.530
Bim A2eT-I2dM	163	-788.258
Bim A2eT-I3dL	145	-803.307
Bim F99E	147	-733.489
Bim F99Q	176	-728.941
Bim FA1	289	-870.386
Bim FD1	163	-839.00
Bim FD2	104	-819.647
Bim FW1	45	-662.237
Bim I86E	100	-833.465
Bim I95A	137	-821.970
Bim Q90E	164	-802.245
Bim XXA1	165	-898.154
Bim XXA1 G2gE	10	-862.512
Bim XXA1 K3bR	16	-900.109
Bim XXA1 Y2dI	54	-679.311
Bim XXA1 Y4eK	229	-915.794
Bim XXA4	225	-747.859
Bim Y103K	148	-719.026
Bim 2A	246	-754.999
Bim A2eE	113	-734.409
Bim A2eF	231	-775.409
Bim A2eG	226	-735.479
Bim A2eH	263	-844.205
Bim A2eI	259	-887.910
Bim A2eK	264	-842.711
Bim A2eL	77	-874.300

Bim A2eN	215	-678.327
Bim A2eP	202	-694.862
Bim A2eQ	202	-791.765
Bim A2eR	245	-887.375
Bim A2eS	78	-858.248
Bim A2eT	203	-843.496
Bim A2eV	178	-814.917
Bim A2eW	235	-758.449
Bim A2eY	250	-763.778
Bim E2gA	92	-789.584
Bim E2gD	219	-744.218
Bim E2gF	300	-886.209
Bim E2gG	139	-855.656
Bim E2gH	121	-792.691
Bim E2gK	224	-668.707
Bim E2gN	133	-675.042
Bim E2gP	249	-683.154
Bim E2gQ	271	-793.901
Bim E2gR	122	-856.822
Bim E2gS	123	-867.419
Bim E2gT	104	-760.384
Bim E2gW	176	-732.981
Bim E2gY	41	-897.170
Bim EgV	153	-785.276
Bim L3aD	72	-744.157
Bim L3aE	172	-710.478
Bim L3aH	101	-740.375
Bim L3aK	142	-737.470
Bim L3aN	181	-727.323
Bim L3aQ	206	-753.693
Bim L3aR	199	-731.317
Bim L3aS	128	-729.699

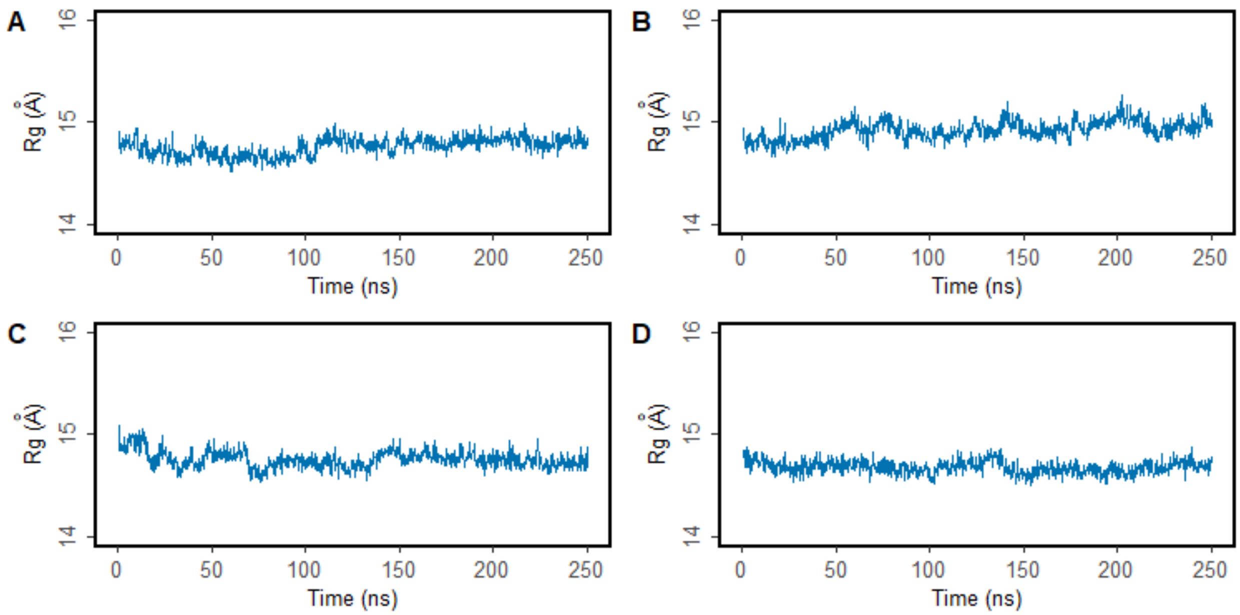

Figure S1. Radius of gyration (Rg) of Mcl-1 protein in 250 ns MD simulations.
(A) Mcl-1/SAH-MS1-18 (B) Mcl--1/E2gI (C) Mcl-1/E2gY, (D) Mcl-1/XXA1 F3dI.

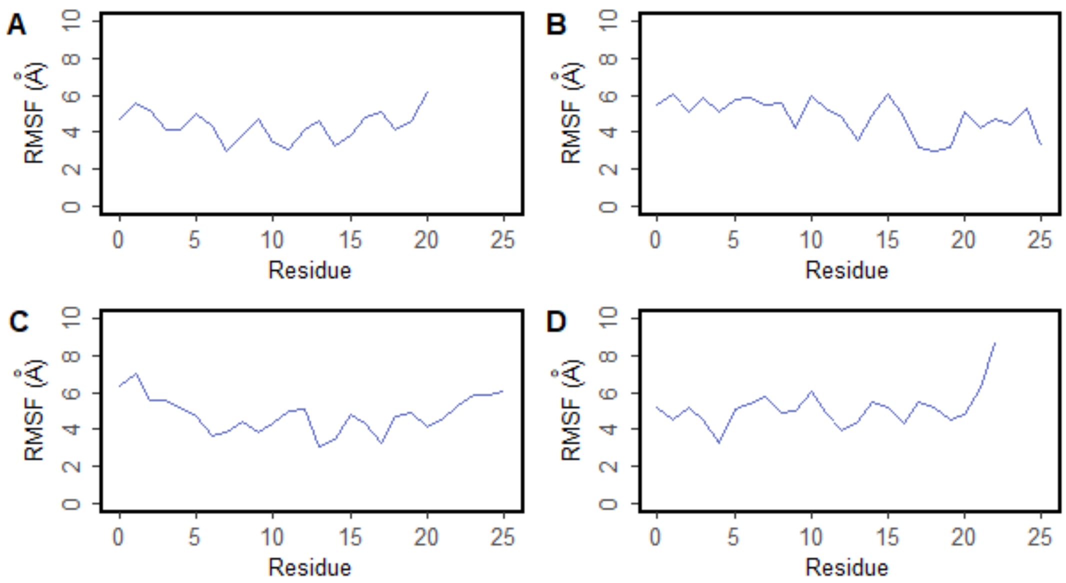

Figure S2. Root Mean Square Fluctuation (RMSF) of bound peptide Cα atoms after aligning the protein structure in each frame of the simulation. (A) SAH-MS1-18, (B) E2gI (C) E2gY, (D) XXA1 F3dI.
